# Technology description, initial experience and first impression of HUGO™ RAS robot platform in urologic procedures in Brazil

**DOI:** 10.1590/S1677-5538.IBJU.2023.9910

**Published:** 2024-02-07

**Affiliations:** 1 Hospital Israelita Albert Einstein Departamento de Urologia São Paulo SP Brasil Departamento de Urologia, Hospital Israelita Albert Einstein, São Paulo, SP. Brasil;; 2 Hospital Israelita Albert Einstein Departamento de Uro-oncologia e Cirurgia Robótica São Paulo SP Brasil Departamento de Uro-oncologia e Cirurgia Robótica, Hospital Israelita Albert Einstein, São Paulo, SP. Brasil;; 3 FMABC Faculdade de Medicina do ABC Disciplina de Urologia Santo André SP Brasil Disciplina de Urologia da Faculdade de Medicina do ABC – FMABC, Santo André, SP. Brasil

## COMMENT

Robotic surgery is a reality in the practice of urologists in developed and in several undeveloped countries. The first clinical procedures were performed in 2000 and since then was observed a fast spread in surgical practice, especially in Urology (1).

The Intuitive company remained exclusive in the market of production of robotic platforms in the past two decades. With the end of patent, different companies are developing new robotic platforms. The market will select which equipment will add real clinical benefits to be incorporated into daily practice (2).

To be able to evaluate a new technology, is necessary to remember the main issues related to the standard robotic platform with impact on the improvement of quality and surgical technique. Among them, it is important to mention (3, 4):

- Surgeon's ergonomic;
- Surgeon autonomy in controlling the camera and others instruments.
- Three dimensions vision;
- Tremor elimination;
- Instruments that allow a wide movement similar to surgeon hands;
- Safety devices.

Is also important quote the issues and limitations of the first platforms that still hinder or preclude them from being implemented in many centers. Among them can be mentioned: high cost, availability for training and qualification.

Thus, for a new technology to be interesting, it must offers the same benefits and supply some needs and limitations not met by other platforms.

The company that is entering in the competition and in this technological race is Medtronic. One of the largest medical device companies in the world produced its first robotic surgery platform called Hugo™ RAS (HR) - Medtronic, Minneapolis, MN, USA.

This platform emerged with the aim of offering a safe tool that allows the surgeons to operate with the same quality as they perform with the "standard" platform.

As a challenge, this technology comes with the proposal of having more accessible cost, allowing access to a greater number of patients and the training of more surgeons.

The "open" console brings the concept that people in training can follow the surgery with the same view of the surgeon, improving the learning curve. Other possible benefit is to be multi-modular with the objective of being more versatile and able to favor "docking" using only the necessary number of arms.

To talk about incorporating a new technology, is necessary remember that there are two sorts of surgeon's profiles. Those who have no experience with robotic surgery and those who are already used to the Da Vinci (DV) platform and in this moment must adapt to a new one.

The aim of this article is to report the results that are already in the literature and our initial experience as well as future perspectives important to highlight.

## LITERATURE REVIEW

The literature about Hugo™ RAS system (Medtronic, Minneapolis, MN, USA) and Urology is still scarce due to the fact that the first surgeries with this platform started in 2021.

The initial studies proposed to prove the safety and functionality of this platform in performing oncological and non-oncological urological surgeries.

Among the uro-oncologic surgeries, the radical prostatectomy is the urologic surgery most performed worldwide. In this direction, it is also the most discussed topic at articles. The use of HR to treat prostate cancer appears to be a safe technique, without losing agility and maintaining satisfactory perioperative results (5-8).

The same feasibility was identified when performing adrenalectomies and nephrectomies. Despite these studies being observational and with few patients included, the initial results described generate an inference that the using the HR the oncologic results are satisfactory and maintains the perioperative results obtained with the standard platform (9-11).

The application of the new robot system is not restricted to the cases already mentioned, studies have also shown a role in non-oncological surgeries such as simple prostatectomy, sacropexy and others (12-14).

### Description and characteristics of the new platform

For the adoption and adaptation with a new platform, it is of fundamental importance for the surgeon to have detailed knowledge of the platform and its resources, as well as its limitations.

It is important to highlight that before the start of the transition to the new platform, our team already had the experience of more than 2,000 procedures with the "Da Vinci" platform and, therefore, it is natural to compare different parts of each equipment with the other.

**Figure 1:**
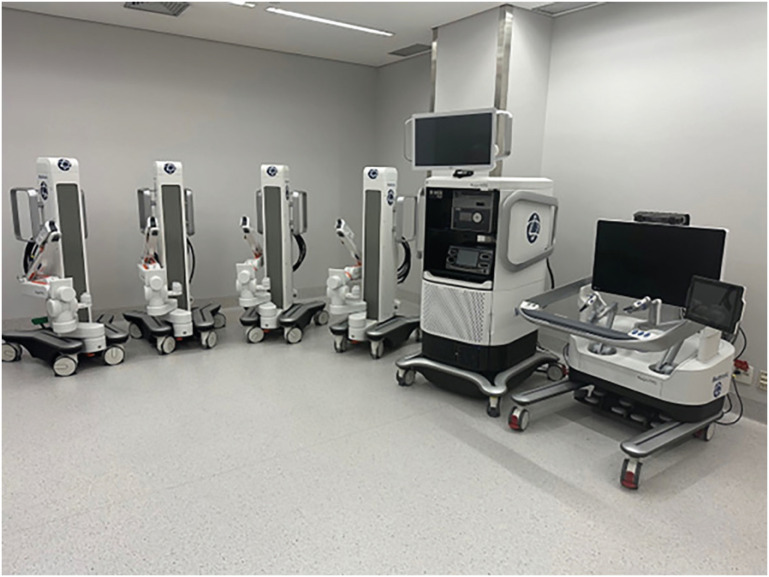
Complete system with surgeon console, main tower, and modular Arms carts (from the right to left).

The HR robot consists of a surgeon console, modular Arms carts and a main tower (Figure-1).

### Console

#### 3D screen

In the DV robot, the surgeon is "immersed" in the console and the surgeon needs to remove the head from the console to have visual contact with the operating room.

In HR, the surgeon looks at a "television screen" with 3D glasses and, with that, it is possible to follow the team's movement.

It is important to highlight that the surgeon's glasses have a sensor that, if the surgeon looks to the sides, the arms are locked for safety. This feature of the HR has the advantage of allowing other people to follow the surgery with the same view of the surgeon and allows the surgeon to keep informed about everything that is happening around, however, the great disadvantage is that external factors can disturb the concentration of surgeon.

**Figure 2:**
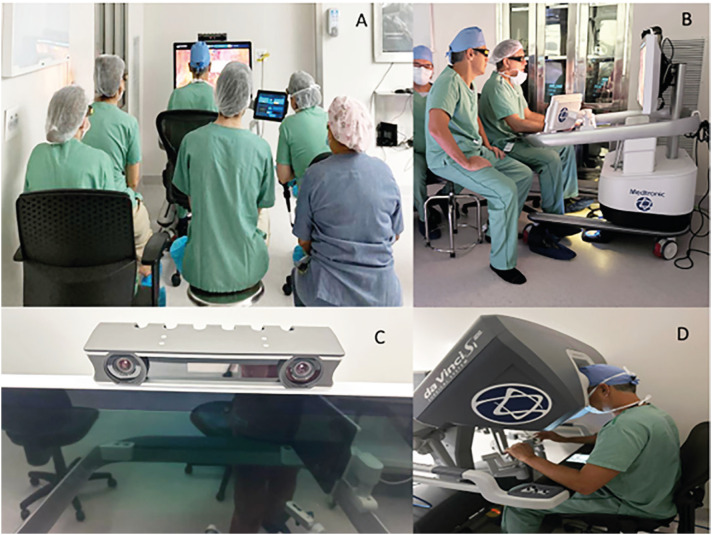
A) Fellows and students watching the surgery with the same 3D view of the surgeon; B) HUGO™ RAS console, pay attention to the surgeon's ergonomics; C) Sensor on top of the screen that, when the surgeon removes his face, the arms stop working, D: DV Console, pay attention to the surgeon's ergonomics.

Our impression is that this console allows a better ergonomic position because is possible to sit more comfortably. In DV the surgeon has to lean a little forward to have a great view of the screen (Figure-2).

For some surgeons the open console was strange and more challenging because the frequency of movements in the room distracts the surgeon attention. However, in our case to avoid this, we placed the monitor facing the wall and request that as few people as possible circulate in the operating room during the procedure. The image quality is excellent, the major difference is that we do not have the feeling of being immersed and the image is a little further away, in our view this does not affect the performance of surgeries.

#### Pedal

**Figure 3:**
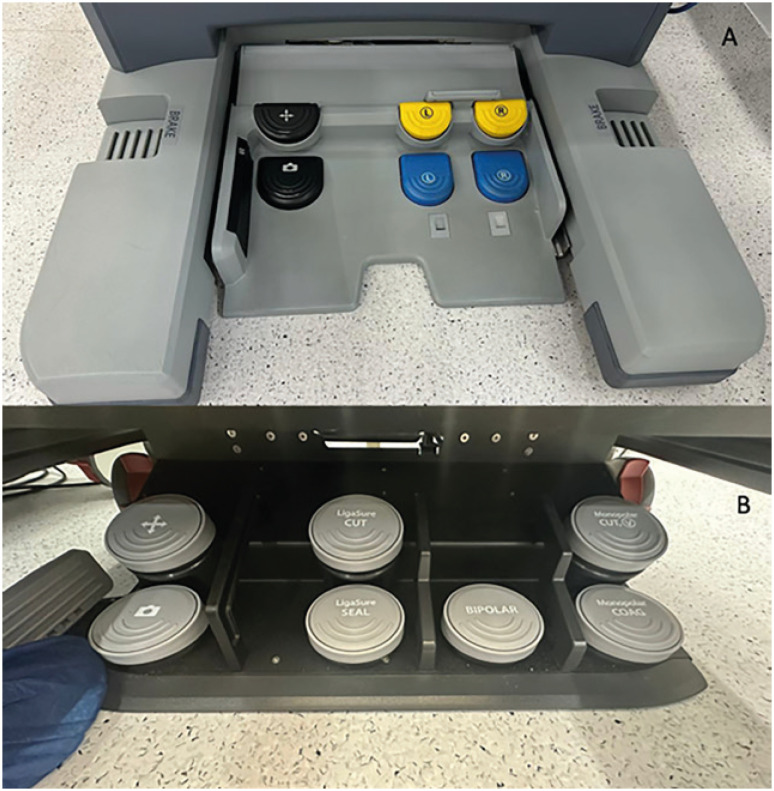
A) Pedal of Da Vinci; B) Pedal of Hugo™ RAS.

The Pedal is similar to the DV containing additional pedals for future harmonic instruments implementation. In contrast with DV, the HR pedals have some safety mechanisms for activation energy and changing the arm in movement (Figure-3).

For both monopolar and bipolar power activation, it is necessary to initially press the pedal for 1.5 seconds, release it and then activate it again. If the energy pedals remain for more than 30 seconds without being used, a new activation will be necessary. In the same way is the instruments swap pedal; in the DV one click is enough and the arm in use changes, in the HR it is necessary to keep it pressed for 1.5 seconds for the change occurs.

Initially these issues did not please us, as it delayed the procedure since we often did not activate it. However, with the passing of the procedures, activation became something natural.

We believe that, for surgeons at the beginning of the learning curve, this is an important safety mechanism, as we often experience surgeons in training inadvertently activating the energy pedals, however, we believe that it may be an option that could be activated according to the surgeon preference and necessity.

#### Manual control

**Figure 4:**
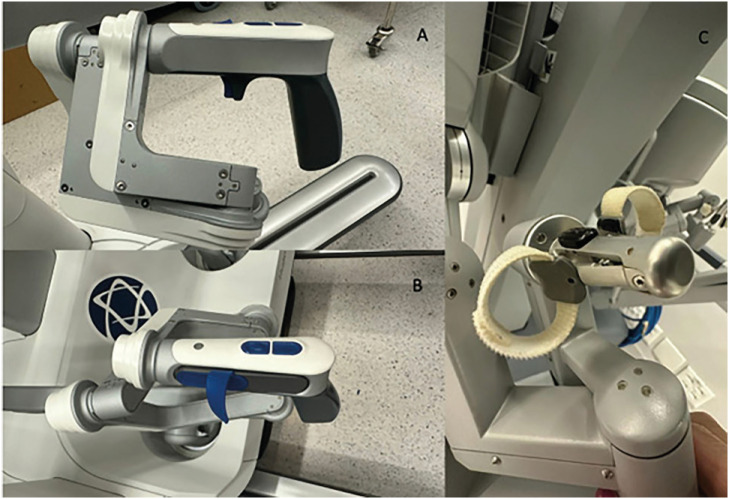
A) Manual control of Hugo™ RAS (right hand); B) Manual control of HUGO™ RAS (left hand); C) manual control of Da Vinci.

**Figure 5:**
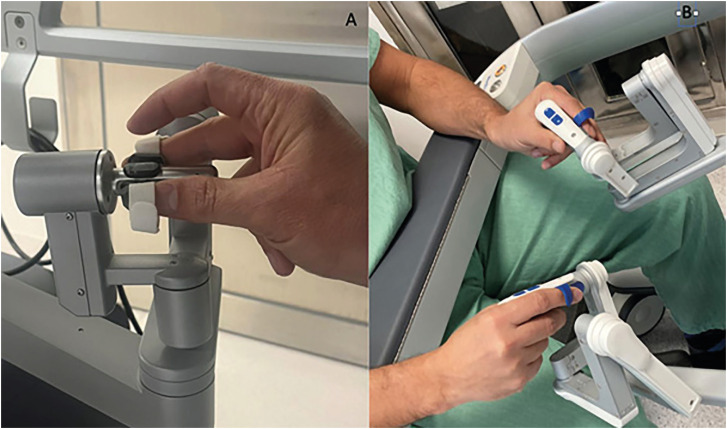
A) manual control of Da Vinci; B) Manual control of Hugo™ RAS.

HR hand control has a different format than DV. In HUGO™, we hold it as if it were a "revolver handle", we work with the index fingers and the first finger, and the third finger is used for the "Clutch" (Figure-4 and Figure 5).

The "trigger" button has two functions. A superficial grip locks the arm, and a deep grip performs the "clutch", that is, the surgeon moves his hands to have poor ergonomics without the tweezers moving inside the patient. This double function is one of the functions that we least like about this console, because often we just touch it superficially and the arm locks, and the idea was to make a clutch. But we also observed that with more cases performed these events became more infrequent.

#### Modular arms

The HR has modular arms carts. In this perspective, the surgeon can decide how many arms are needed for each type of procedure. The arms have a wide range of motion and can be placed in a very versatile way.

**Figure 6:**
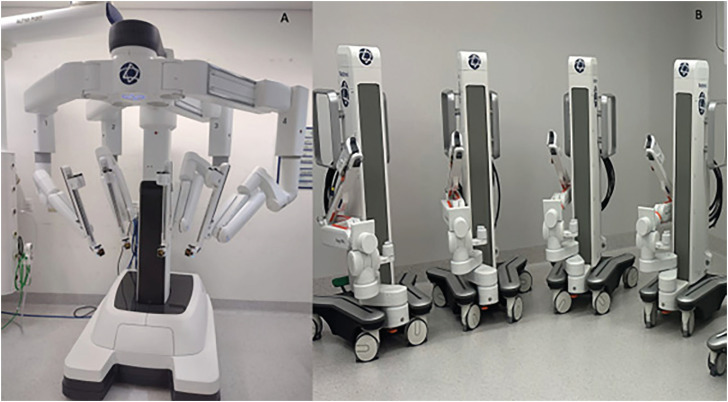
A) Da Vinci Xi arms; B) Hugo™ RAS Modular arms.

Initially, we believed that, as it is multi-modular, it would occupy less space in the operating room, however the arms are still large with a robust base and takes up more space than DV (Figure-6).

**Figure 7:**
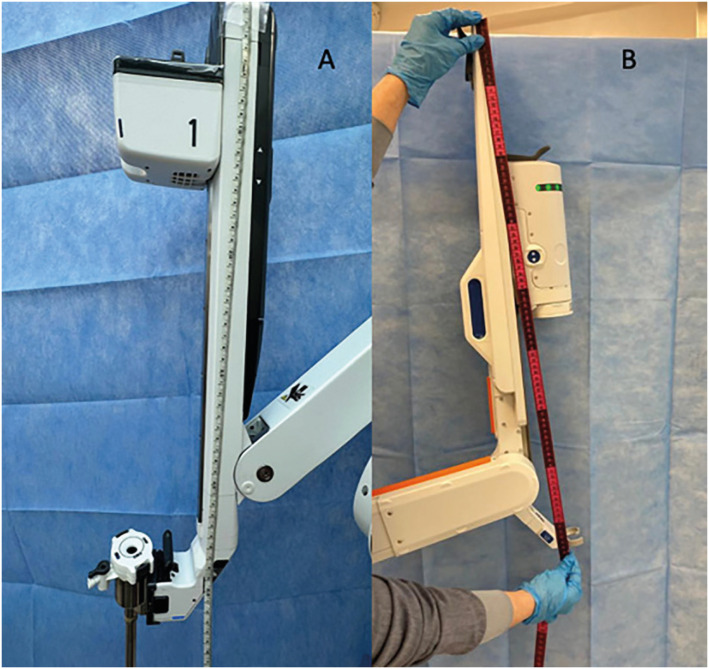
A) Da Vinci Xi arm length; B) Hugo™ RAS Modular arm length.

The arms of the HR have 85 cm, bigger than those of the DV that have 53 cm (Figure-7). These longer arms make it a little more difficult for the assistant to manipulate and are more susceptible to collisions during the procedure.

This is an important point of attention because the assistant always works in a more uncomfortable way in relation to the DV and he needs to be attentive to robot arm does not collide with his body.

#### Instruments

**Figure 8:**
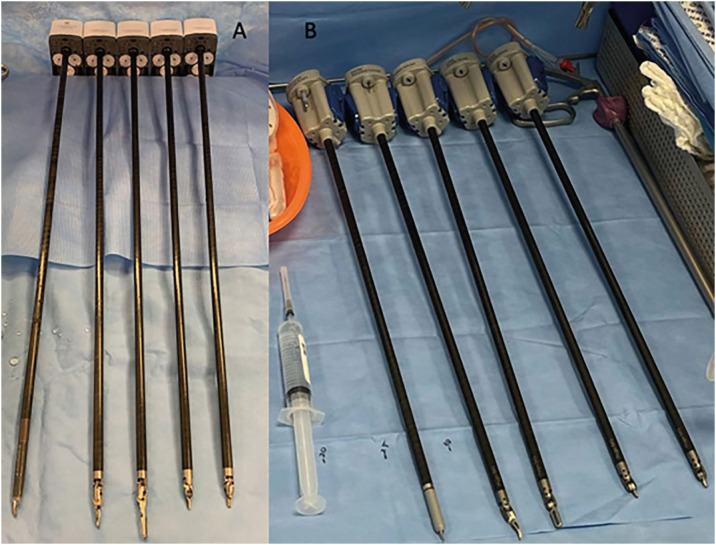
A) Table with Da Vinci instruments; B) Table with HR instruments

The HR instruments are shorter than DV instruments (Figure-8).

**Figure 9:**
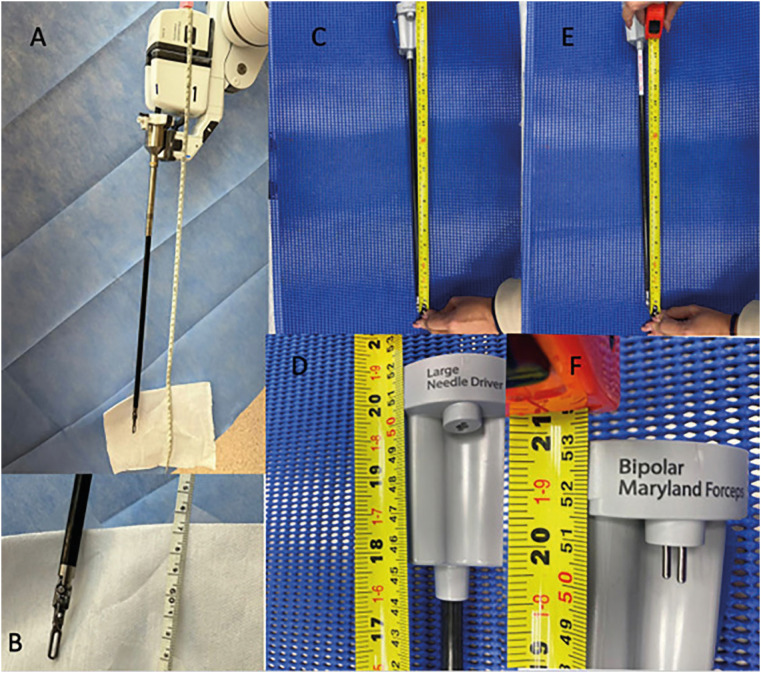
A and B) DV prograsper total length; C and D) HR large needle driver total length; E and F) HR bipolar maryland forceps total length.

HR Maryland forceps has 53 cm and HR large needle driver has 52 cm. The longest HR instrument commonly used in urology is the double fenestrated with 54.3 cm total length. DV Maryland forceps and large needle driver has, respectively, 62 cm total and 61 cm total size. The longest DV instrument used in urology is prograsper with 63 cm (Figure-9).

The instruments disposable in Hugo™ RAS are: scissors, fenestrated bipolar, Maryland bipolar, large needle driver, extra-large needle driver, Cadiere forceps, Cadiere secure, double fenestrated and toothed claw.

The needle driver has an excellent gripping force, and the system has a possibility to set a "2 times" rotation. This was a very interesting characteristic that facilitated the performance of the vesicourethral anastomosis. There are two kinds of needle driver: the large and the extra-large.

Scissors are more delicate and sharper, however, at the moment they are still single-use and every 40-50 minutes you have to change a new one. At first, we found it odd and worried about the cost. But the company does not charge extra for this.

The great advantage is that we operate all the time with excellent quality scissors. When we close the scissors, they give a little bounce. At first it seemed strange but during the procedure it did not impact safety and quality. But it is certainly a relevant point for the company to improve.

Regarding the traction instruments, it is important to highlight that the Cadiere forceps does not have a great traction capacity, making some steps of the procedure difficult, being more recommended to use the "cadiere secure" or the "toothed".

To perform urology surgeries the instrument from DV most missed was the Tenaculum to traction the prostate adenoma during simple prostatectomy. To remedy the lack of this instrument, the surgical assistant needed to use laparoscopic toothed gripping forceps to help trace the adenoma during the procedure, which we have as a weakness as it impairs the speed and autonomy of the surgeon.

#### Main Tower

The main tower is very similar to the DV Xi. The components are a HD screen, a Karl Storz Imagem system and a Valleylab energy platform.

The image generated by Storz shows a brilliant tridimensional high-definition view and helps to identify the correct surgical plans during the procedure.

The endoscope can be placed in each one of all arms carts. This point can be extremely helpful during performing a surgery with steps in different quadrants.

## DOCKING

At first moment, as it was a multi-modular platform, we believed that docking could be made easier. However, we believe it is one of the biggest challenges in daily practice.

For each module, a precise positioning in relation to the table is required. Each arm is attached respecting two angles. The first is the tilt angle - inclination of the operative arm compared to the operative bed. The second angle is between the robotic arm and the head of the patient.

**Figure 10:**
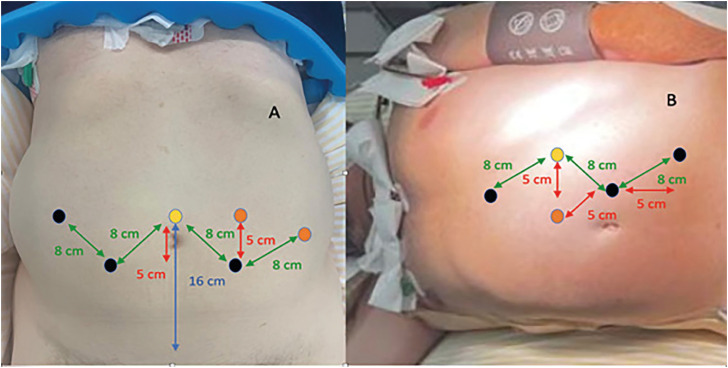
A) Medtronic recommendations for prostatectomy. The endoscope trocar (yellow circle) is placed 16 cm or less from the target. The right arm trocar is placed in a line 5 cm bellow the endoscope line and need stay at least 8 cm distant from the endoscope (black circle). The left arm trocar follows the same instructions of right arm in contralateral side (black circle). The fourth arm of surgeon is placed in the same line of endoscope trocar, with 8 cm or more distant from the right arm. The assistant trocar (orange circle) needs to be placed at least 5 cm distant from the left arm. All trocars need to stay at least 2 cm from bone prominence; B) Medtronic recommendations for left nephrectomy. The endoscope trocar (yellow circle) is placed at least 5 cm from the line where will be placed the left and right arm of surgeon. At least 5 cm of distance between the fourth arm trocar and right hand of surgeon. The robotic trocars need to be placed at least 8 cm distant from each other. The assistant trocar should be placed medial to the left and right arms and near the median line of patient, at least 5 cm from the other trocars.

**Figure 11:**
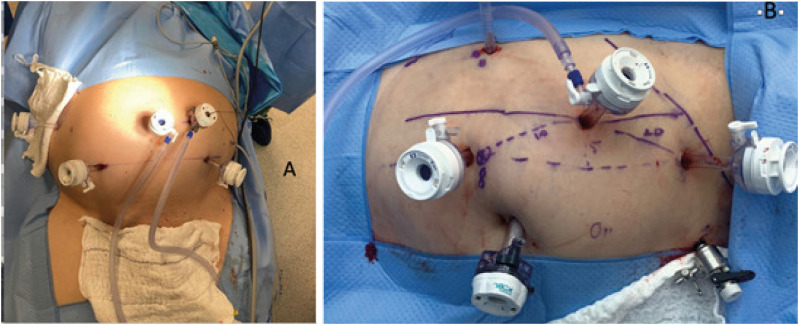
A) Image of trocers placement - radical prostatectomy with Hugo™ RAS; B) Right parcial nephrectomy with Hugo™ RAS.

The Medtronic recommends angles for each arm according to the surgery and add that alterations may be necessary according to the patient's body type, the patient's pathology or the surgeon's preference. (Figure-8) In the first cases we try to reproduce exactely what the suggested regarding the docking, trocater's placement and patient suggested position. But in few cases, we realized that, to reproduce our technique some modification must be performed (Figures 9-11). Important to point out is that the angle of the arms can be adjusted according to surgeon preference without compromise the functioning of the arms carts.

## SECURITY

The HR system has many more safety features than the DV. When operating with the HR, the team must get used to triggering different alarms.

The goggle sensor and the pedal drive system make the procedure safer, especially for surgeons at the beginning of the learning curve.

It is important to highlight that, so far, we have not experienced any adverse events with the platform, and we believe that it is a technology that follows all the necessary protocols to perform safe robotic surgery.

## SURGICAL TECHNIQUE

In radical prostatectomy, it was possible to perform the same standard of surgical technique since the first procedure, making it possible to perform surgery with the same technical rigor used in surgeries with the DV. It is only necessary to adapt it to the assistant's portal.

Regarding partial nephrectomy, we believe that we will need more time for adaptation, the concept of trocar placement changes a lot in relation to the DV. These modifications are necessary to reduce conflict. In this procedure, we found it very difficult for the assistant to carry out some steps, which made it difficult at times for an adequate presentation. The procedures with HR were uneventful, respecting all oncological principles. However, we believe that it is still necessary to improve the standard placement of trocars to facilitate the performance of a procedure with the same standards as the DV.

## SIMULATOR AND PERMISSION TO USE

The HR has a simulator with some tasks like manipulation of needle, using of instruments, suture exercises and others. The repetitive exercises in simulators reduce the time necessary in dry and wet lab. For new robotic surgeons it allows get familiar with the platform and acquire a proficiency in some robotic activities.

The quality of simulator and exercises presented is excellent and similar to DV.

About the permission to surgeon use, Medtronic suggests the Hugo™ RAS should be used by medically trained surgeons with a full understanding of the safe operation of the system, in accordance with the hospital's credentialing policies regarding the use of new equipment. This robot access policy is more flexible and comprehensive compared to DV, delegating to the hospital the function of releasing qualified surgeons for the procedure.

## LIMITATIONS AND FUTURES PERSPECTIVES

Some limitations of the Hugo™ platform must be considered here, for example, the lack of glasses and 3D vision to assistant in the surgical field. The 3D glasses for the assistant would allow better image quality and approximation between the surgeon's and assistant's vision. The absence of some auxiliary resources for more complex surgeries, such as firefly with indocyanine green (fluorescence capability that uses near-infrared technology) and the use of robotic ultrasound, which are present on the Da Vinci platform and not on the Hugo™ RAS, is a limitation of the new robotic model.

Indocyanine green is most used in urological surgeries for complex partial nephrectomies - large renal tumors or with multiple renal arteries - and also for vascularization assessment during neobladder surgery. In other specialties such as gynecology, this feature is also important, for example, for identifying sentinel lymph nodes.

Robotic ultrasound on the Da Vinci platform allows combined intrabdominal vision and ultrasound, helping in various situations such as demarcation of tumor boundaries during complex partial nephrectomies.

## CONCLUSIONS

HR has more safety and training features for new surgeons without previous experience with robotic surgery.

The surgeon may realize a training with the simulator until be comfortable with the console and realizing automatic and natural movements. At least 8 hours in simulator training Is suggested.

The surgeon training is so important like the team training. A team motivated and familiarized with the robotic assisted system and docking is extremely important to the success of the platforms transition.

For surgeons with experience with the DV, the transition seems to be friendlier in radical prostatectomy, however, for partial nephrectomy, the transition is more challenging, and the team must already have experience with the new platform.

We believe that Medtronic could improve the software to make it more personable. The activation of the energy, the double function of the trigger button and the necessity of keep holding the bottom to change the arms, could be a configuration option according to the preference of the surgeon. Others features that need to be developed are 3D glasses for the surgical assistant, firefly with indocyanine green and robotic ultrasound with vision for the console surgeon.

The size of the Arms module must be reduced the size of the arm as well to facilitate the docking and bed side assistant work.

We need a larger casuistic to be able to construct more solid considerations about this new robotic platform.
